# Perinatal exposure to the fungicide ketoconazole alters hypothalamic control of puberty in female rats

**DOI:** 10.3389/fendo.2023.1140886

**Published:** 2023-04-03

**Authors:** Delphine Franssen, Hanna K. L. Johansson, David Lopez-Rodriguez, Arnaud Lavergne, Quentin Terwagne, Julie Boberg, Sofie Christiansen, Terje Svingen, Anne-Simone Parent

**Affiliations:** ^1^ Neuroendocrinology Unit, GIGA Neurosciences, University of Liège, Liège, Belgium; ^2^ National Food Institute, Technical University of Denmark, Kgs. Lyngby, Denmark; ^3^ GIGA-Bioinformatics, GIGA Institute, Université de Liège, Liège, Belgium; ^4^ Department of Pediatrics, University Hospital Liege, Liege, Belgium

**Keywords:** endocrine disrupting chemicals (EDCs), hypothalamus, puberty, transcriptome, reproduction

## Abstract

**Introduction:**

Estrogenic endocrine disrupting chemicals (EDCs) such as diethylstilbestrol (DES) are known to alter the timing of puberty onset and reproductive function in females. Accumulating evidence suggests that steroid synthesis inhibitors such as ketoconazole (KTZ) or phthalates may also affect female reproductive health, however their mode of action is poorly understood. Because hypothalamic activity is very sensitive to sex steroids, we aimed at determining whether and how EDCs with different mode of action can alter the hypothalamic transcriptome and GnRH release in female rats.

**Design:**

Female rats were exposed to KTZ or DES during perinatal (DES 3-6-12μg/kg.d; KTZ 3-6-12mg/kg.d), pubertal or adult periods (DES 3-12-48μg/kg.d; KTZ 3-12-48mg/kg.d).

**Results:**

Ex vivo study of GnRH pulsatility revealed that perinatal exposure to the highest doses of KTZ and DES delayed maturation of GnRH secretion before puberty, whereas pubertal or adult exposure had no effect on GnRH pulsatility. Hypothalamic transcriptome, studied by RNAsequencing in the preoptic area and in the mediobasal hypothalamus, was found to be very sensitive to perinatal exposure to all doses of KTZ before puberty with effects persisting until adulthood. Bioinformatic analysis with Ingenuity Pathway Analysis predicted “Creb signaling in Neurons” and “IGF-1 signaling” among the most downregulated pathways by all doses of KTZ and DES before puberty, and “PPARg” as a common upstream regulator driving gene expression changes. Deeper screening ofRNAseq datasets indicated that a high number of genes regulating the activity of the extrinsic GnRH pulse generator were consistently affected by all the doses of DES and KTZ before puberty. Several, including MKRN3, DNMT3 or Cbx7, showed similar alterations in expression at adulthood.

**Conclusion:**

nRH secretion and the hypothalamic transcriptome are highly sensitive to perinatal exposure to both DES and KTZ. The identified pathways should be exploredfurther to identify biomarkers for future testing strategies for EDC identification and when enhancing the current standard information requirements in regulation.

## 1 Introduction

Age distribution of pubertal signs in humans has been changing over the last decades. It is characterized by an extended distribution towards earliness for initial pubertal stages and towards lateness for final pubertal stages (1). This phenomenon is concomitant with an increased incidence rate in reproductive disorders (1, 2). Such rapid trends suggest that environmental factors play a causal role. Indeed, the programming of pubertal maturation and fertility is finely tuned by sex steroids and highly sensitive to environmental factors (3, 4). A growing body of evidence points towards an association between exposure to endocrine disrupting chemicals (EDCs) and recent changes in pubertal timing or reproductive health (5). While initial studies reported an impact of such environmental factors on male fertility (6), epidemiological data, together with animal models, now indicate that female reproductive health is also sensitive to environmental chemicals (7–10). Epidemiological studies reported associations between prenatal or childhood exposure to estrogenic EDCs such as dichlorodiphenyltrichloroethane (DDT) or bisphenol A with early or late puberty in girls (11–13). Impaired fecundity or premature menopause have also been reported after exposure to diethylstilbestrol (DES) (14, 15), PCOS after exposure to bisphenol A (16), and premature ovarian failure after exposure to perfluoroalkyl chemicals (17). Following such observations, initial animal studies focused on modeling effects of developmental and adult exposure to estrogenic EDCs on pubertal development, ovulation, and fertility. Estrogenic EDCs such as DES, bisphenol A and dichlorodiphenyltrichloroethane have been shown to alter the timing of vaginal opening or first estrus (18–22); reviewed in (1) and disrupt the preovulatory luteinizing hormone (LH) surge, ovarian follicle maturation and fertility in rodents (23–25). In addition, steroid synthesis inhibitors such as phthalates and the fungicide ketoconazole (KTZ) which are known to alter male reproductive function (26–28), have been recently described to impair female reproductive health (29, 30).

We recently showed that perinatal exposure to KTZ and DES delays vaginal opening in female rats without affecting bodyweight (21). This opened up the question of possible effects on central regulation of puberty onset. In the present study, we investigate the brain from the same animal litters to explore hypothalamic mechanisms potentially involved in the disruption of puberty. The reproductive organs have long been considered the major target of EDCs, but brain structures such as the hypothalamus, also appear to be highly sensitive to EDCs (31). GnRH neurons regulate all aspects of reproduction through their pattern of release. The secretory activity of GnRH neurons depends on trans-synaptic and glial inputs mediated by neurotransmitters and cell-cell signaling molecules produced in the preoptic area (POA) and the mediobasal hypothalamus (MBH) (32). New methods have provided more information regarding the transcriptional regulation of the hypothalamic network coordinating reproduction (33–35). This tightly organized network controlling GnRH secretion throughout development appears to be exquisitely sensitive to EDCs (10, 36).

KTZ is a first-generation antifungal imidazole known to disrupt steroid hormone synthesis by inhibiting various cytochrome P450 (CYP) enzymes (37–39). Recent data showed that KTZ, at low concentration (0.041-1.2 μM) induced an inhibition of CYP17A1 as it led to an accumulation of progestagens and corticosteroids and a decrease in androgens and estrogens. At higher concentrations, KTZ suppressed all steroids synthesis (40). DES is a synthetic estrogen (41–43). Its effects appear to be mediated by ERα as mice deficient in ERα (ERαKO) do not develop genital anomalies observed in wild-type mice after DES exposure (44–47). DES activate MAP kinase and PI3K pathways and induce ERK phosphorylation (48, 49).

Although deleterious effects of EDCs on reproductive function are now well recognized, current regulations are still insufficient for identifying windows of sensitivity and effects on female reproductive health (50). Better test methods to identify EDCs are necessary and require a better understanding of modes of action as well as the identification of biomarkers. To address this issue, this study aimed to determine the influence of two EDCs with different modes of action on the hypothalamic transcriptome governing GnRH release.

## 2 Methods

### 2.1 Chemicals

Ketoconazole (KTZ - CAS no. 65,277-42-1, purity 98%) was purchased from BOC Sciences Inc. (USA), and diethylstilbestrol (DES- CAS no. 56-53-1, purity ≥ 99%) from Sigma/Aldrich (cat.no. D4628). To use as control and vehicle, corn oil was purchased from Sigma/Aldrich (cat.no. C8267).

### 2.2 Animals and experimental design

**Figure 1:**
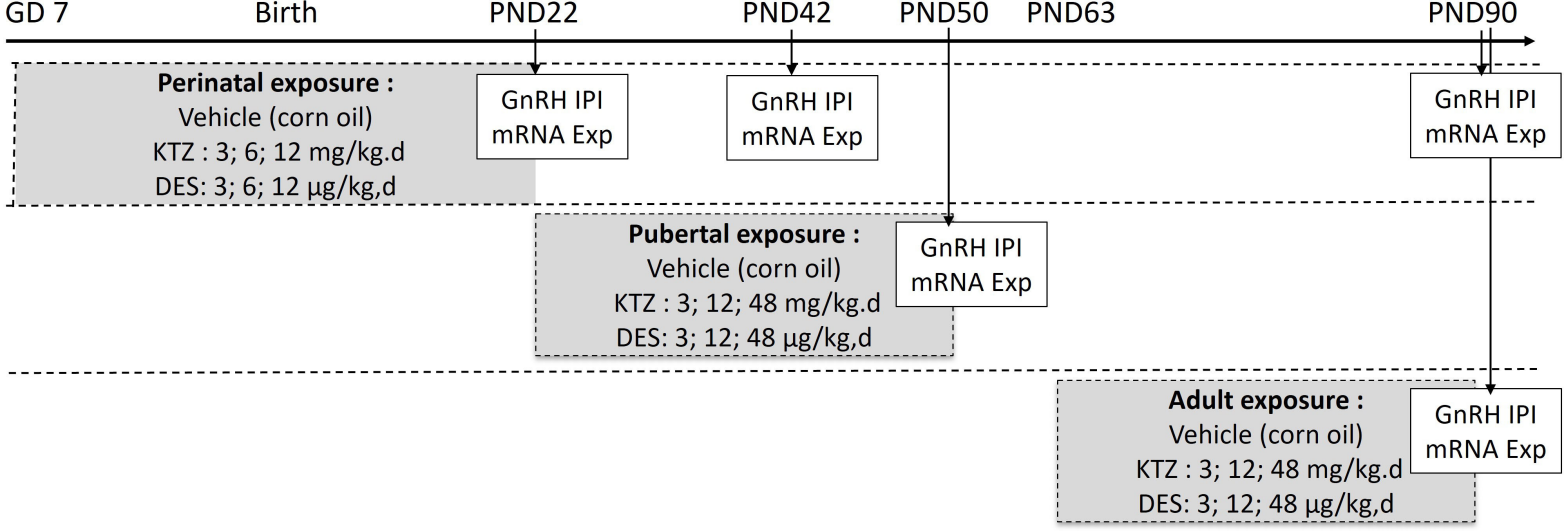
Exposure protocol. Rats were exposed to vehicle or diethylstilbestrol (DES) or ketoconazole (KTZ) during the perinatal period (gestation day (GD) 7 to postnatal day (PD) 22), pubertal period (PD22 to PD50) or adulthood (PD63 to PD90). Arrows indicate the age at which GnRH secretion and hypothalamic mRNA studies were conducted. GnRH IPI, GnRH interpulse interval; mRNA Exp, mRNA expression.

Housing and exposure of the rats were performed in the National Food Institute facilities, Technical University of Denmark (Lyngby, Denmark). Ethical approval was obtained from the Danish Animal Experiments Inspectorate under the authorization number 2015-15-0201-00553. The National Food Institutes In-house Animal Welfare Committee for animal care and use oversaw the experiments. The animals were housed in High Temperature Polysulfone (PSU) cages with Tapvei wooden shelters. The cages were placed in ScanTainers (Ventilated Cabinets from Scanbur) with controlled environmental conditions: 12 h light (21.00 - 9.00 h): 12 h dark (9.00–21.00 h) cycle, humidity 55% ± 5, temperature 22°C ± 1°C and ventilation changing air 50–60 times per hour. Animals were fed Altromin 1314 (soy and alfalfa free) and tap water (BPA free bottles 84- ACBT0702SU; Polysulfone 700 mL w/ring square) ad libitum. As illustrated in **Figure 1**, three different exposure scenarios were used: *perinatal, pubertal*, and *adult*. For perinatal exposure (21), time-mated nulliparous Sprague-Dawley rats (CD IGS Rat, Crl : CD(SD), Charles River Laboratories, Sandhofer Weg 7, Sulzfeld, Germany) were supplied on gestation day (GD) 3. The day when a vaginal plug was detected was designated GD 1. On GD 4, animals were pseudo-randomly distributed into seven groups with similar body weight distributions. Dams were exposed by oral gavage with either chemical or vehicle once daily from GD 7 until birth and from the day after birth until postnatal (PND) 22. Doses for DES were 0; 3; 6; 12 µg/kg bw/day and doses for KTZ were 0; 3; 6; 12 mg/kg bw/day. For pubertal and adult exposure, females were exposed by oral gavage with either chemical or vehicle once daily from PND 23 to 49-52 or from PND 63 to 91-94, respectively. The exact day of study termination differed in a four-day interval to enable dissection in the di-estrous stage. The doses for DES were 0; 3; 12; 48 µg/kg bw/day and the doses for KTZ were 0; 3; 12; 48 mg/kg bw/day.

### 2.3 Hypothalamic explant incubation and GnRH assay

In order to assess the effect of DES or KTZ exposure on GnRH frequency, GnRH interpulse interval was measured using a hypothalamic explant incubation system followed by a GnRH radioimmunoassay, as described previously (51, 52). For the perinatally exposed animals, hypothalamic explant incubation was conducted for low and high dose groups of both DES and KTZ on PND 22, 42 and 90. For animals exposed during pubertal and adult periods all doses of DES and KTZ were included and hypothalamic explant incubation conducted on PND 50 and 90 respectively. Briefly, after decapitation, brains were collected to dissect the hypothalamus. Hypothalamic explants were transferred into an individual chamber, in a static incubator, submerged in MEM. The *ex vivo* explants incubation chamber contained a water-saturated atmosphere of O_2_ at 37.5°C. Incubation medium was then collected and renewed every 7.5 min for a period of 4 hours. The GnRH released into the incubation medium was measured in duplicate using a radioimmunoassay method as described previously (53). In short, samples were preincubated with CR11-B81 (AB_2687904) rabbit anti-GnRH antiserum (initial dilution 1:20,000) (provided by Dr.V.D.Ramirez, Urbana, Illinois) during 24h at 4°C. GnRH labeled with ^125^I (30,000 CPM) and rabbit serum (dilution 1:100) were added for 24h at 4°C. Finally, precipitation was induced by a solution of sheep anti-rabbit antiserum (dilution 1:200; CER Groupe), polyethylene glycol (60g=L), tween, and cellulose. Radioactivity was counted on a gamma-counter (Wallac CliniGamma). The intra-assay and inter-assay coefficients of variation were 7% and 10% and the limit of detection was 5pg/100 µl.

### 2.4 Hypothalamic mRNA extraction

Expression of hypothalamic genes in females was analyzed by RNA-seq after perinatal exposure (at PND22, PND42 and PND90) and by real-time RT-PCR after pubertal and adult exposures. After decapitation, the pre-optic area (POA) and the mediobasal hypothalamus (MBH) were rapidly dissected and frozen. The brain was placed ventral side up. The dissection began by two sagittal incisions along the lateral hypothalamic sulci. Two transversal incisions were made 2 mm ahead from the anterior boundaries of the optic chiasm and along the caudal margin of the mammillary bodies. Finally, a frontal incision was made under the ventral surface of the hypothalamus. Total RNA was extracted from the MBH and POA using Universal RNA mini kit (Qiagen,Venlo, Netherlands) following the manufacturer’s instructions.

### 2.5 RNA sequencing

RNA-seq analysis was carried out on total RNA extracted from MBH and POA of female rats at PND22, PND42 and PND90 after perinatal exposure to vehicle, DES (3; 6; 12 µg/kg bw/day) or KTZ (3; 6; 12 mg/kg bw/day) (n = 5/each group; 210 samples in total). Library preparation and sequencing were performed at the GIGA Genomics facility (University of Liège, Belgium). RNA integrity was verified on the Agilent Bioanalyser with RNA 6000 Nano chips, RIN scores were > 7.5 for all samples. Illumina Truseq stranded mRNA Sample Preparation kit was used to prepare libraries from 1 microgram of total RNA. Libraries were quantified by qPCR with the KAPA Library Quantification Kits for Illumina^®^ platforms. Sequencing was performed on Illumina^®^ NovaSeq™6000 Sequencing System. The average read-depth of the data is ~25-30M reads. The data were processed through the nf-core “rnaseq” pipeline version 1.4.2 (https://nf-co.re/rnaseq/1.4.2). The quality control of the samples was assessed with FastQC software v0.11.8 (https://www.bioinformatics.babraham.ac.uk/projects/fastqc/). Reads were aligned on the Rattus Norvegicus genome, using Rnor_6.0 genome build and annotations from the Ensembl release v102 (ensembl.org) using STAR software v2.6.1d (https://github.com/alexdobin/STAR). Gene expression was determined using featureCounts v1.6.4. QCs of mapping and quantification were assessed with ~80-85% of mapping rate and ~65-70% of assignation rate. Raw data were deposited on the GEO repository under accession number GSE225359.

### 2.6 RNA-seq data analysis

The count matrix was imported in R environment and analyzed using the *DESeq2* package (54). Differentially Gene Expression Analyses were performed by pairwise comparisons using classical *DESeq2* methods, and differentially expressed genes were selected using an adjusted p-value (FDR) lower than 0.05 and no threshold on the log2 Fold Change (Log2FC). Samples were clustered using Principal Component Analysis while we used Venn Diagram (using ggVennDiagram) to cross results from the different groups.

### 2.7 Ingenuity pathway analysis

Datasets generated by the RNA-seq analysis, including log2-fold change and adjusted p-value, were imported into the Ingenuity Pathway Analysis Tool (v01-20-04 version). In IPA, differentially expressed genes (DEG) are mapped to genetic networks available in the Ingenuity database and then ranked by score. The score is generated based on hypergeometric distribution, where the negative logarithm of the significance level is obtained by Fisher’s exact test at the right tail (55, 56). The Z-score >2 was defined as the threshold of significant activation, whilst Z-score <−2 was defined as the threshold of significant inhibition. The Comparison Analysis tool of IPA was used to study canonical pathways and upstream regulators among the different doses of KTZ and DES. The threshold for significance for the heat map of canonical pathways was a Z-score >2. The heatmap were then filtered for “*Neurotransmitters & other nervous system signaling*”, “*Hormone biosynthesis”, “Nuclear receptor signaling”* and *“Growth factor signaling*” representing around 100 canonical pathways.

### 2.8 RT-qPCR

Reverse transcription of 500 nanograms of RNA for each sample (n= 6/group) was performed using the Transcriptor first strand cDNA synthesis kit (Roche, Germany). For real-time semi quantitative RT.PCR reactions (RT-qPCR), FastStart Universal SYBR Green Master (Rox) (Roche, Germany) and a LightCycler 480 system (Roche, Germany) were used. Four microliters of cDNA (previously 10-fold diluted) were added to a mix of 5 μl of SYBR green, 0.3 μl of both forward and reverse primers and 0.4 μl of nuclease-free water. Cycle threshold (Ct) values were obtained from each individual amplification curve and the average Ct was calculated for each target gene in each sample. Quantification of relative gene expression was performed according to the 2−ΔΔCt method, which takes into account reaction efficiency depending on primers (57). All assays had amplification efficiencies between 1.9 and 2.1. β-actin was used as normalizing gene. The primer sequences and information are provided in **Supplemental Table 1**.

### 2.9 Statistics

For the analyses of GnRH interval interpulse and the RT-qPCR, numerical values were expressed as mean ± SEM. As the data followed a normal distribution, a one-way ANOVA followed by a Newman-Keuls multiple test were performed. For the analysis of the RNAsequencing analysis, the DEGs were identified by adjusted p-value >0.05.

## 3 Results

### 3.1 Perinatal exposure to KTZ or DES slows down maturation of GnRH pulsatile secretion

**Figure 2:**
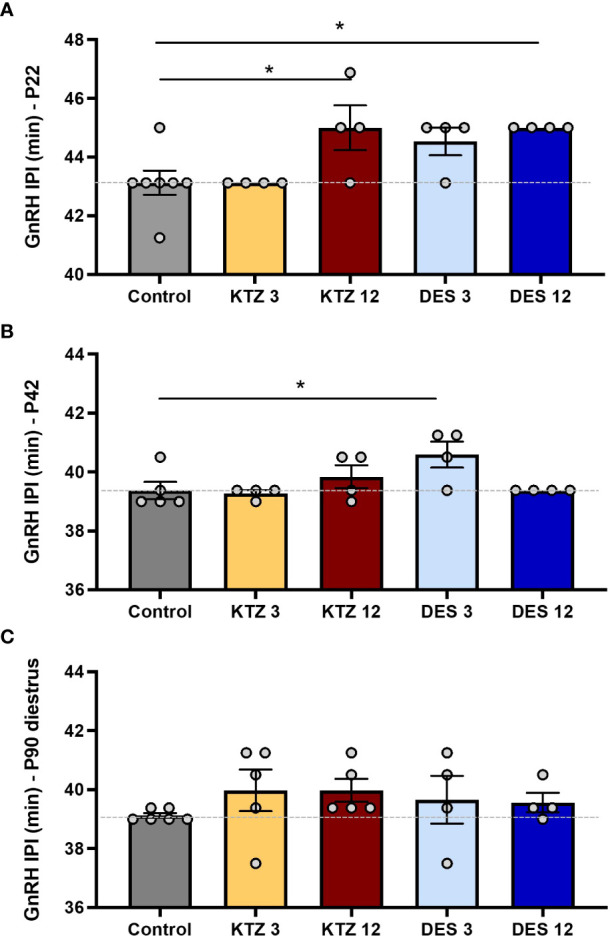
Effects of perinatal exposure to KTZ and DES on pulsatile GnRH secretion from hypothalamic explants *in vitro*. **(A)** GnRH interpulse interval *ex vivo* using hypothalamic explants obtained on PND 22 from female rats perinataly exposed to vehicle (control), DES (3 or 12 µg/kg bw/day) or ketoconazole (3 or 12 mg/kg bw/day). **(B)** and **(C)** GnRH interpulse interval *ex vivo* using hypothalamic explants after perinatal exposure to vehicle, DES or KTZ and obtained on PND 42 and PND 90 respectively. Data are mean (n=4-6) +/- SEM; *p < 0.05 versus control at the same age. The dotted line indicates the mean interpulse interval for the control group.

We previously showed that perinatal exposure to DES or KTZ delays vaginal opening in female rats without affecting bodyweight (21). Because puberty results from the activation of GnRH secretion, this opened up the question of a potential delayed activation of GnRH secretion caused by exposure to DES and KTZ. Pulsatile GnRH secretion from individually incubated hypothalamic explants displays a developmental acceleration between day 5 and day 25, before the onset of puberty (58). Consistently, exposures to EDCs that advance or delay pubertal onset are associated with an acceleration or retardation of GnRH release, respectively (9, 19, 20), indicating the sensitivity of such an assay. We aimed to study the effect of perinatal exposure to KTZ or DES on GnRH secretion on PND22. GnRH interpulse interval (GnRH IPI) was significantly increased by exposure to EDCs (F_4,18_ = 4.727; p=0.0088). *Post hoc* analyses indicated that the exposures to 12 µg/kg/d of DES and 12 mg/kg/d of KTZ significantly increased GnRH interpulse interval at 22 days of age compared to the Control group (p = 0.02 and p = 0.02 respectively; **Figure 2A**), indicating a delay of the maturation of GnRH secretion. This was consistent with the significant delay in vaginal opening reported in the same animals for all doses of DES or KTZ at 6 mg/kg/d (21).

To determine whether the effects of DES and KTZ persisted later in life, we studied GnRH pulsatile release after puberty: at PND42 and during adulthood (diestrus stage) at PND90. ANOVA analysis of GnRH interpulse interval at PND 42 showed a significant effect of exposure (F_4,16_ = 3.312; p=0.0371). *Post hoc* analyses revealed that only the lowest dose of DES (3 µg/kg/d) significantly increased GnRH interpulse interval on PND 42 (p = 0.0301; **Figure 2B**). Alterations of GnRH release caused by exposure to DES or KTZ before puberty did not persist on PND90 (**Figure 2B, C**).

### 3.2 Pubertal or adult exposure to KTZ or DES did not affect GnRH pulsatile secretion

We also aimed at determining whether there was a sensitive exposure window for DES and KTZ effects on GnRH secretion. GnRH pulsatile release was studied immediately after pubertal or adult (diestrus stage) exposure to DES and KTZ. The ANOVA tests did not indicate any significant disruption of GnRH pulsatility after pubertal (F_6.27_ = 0.347; p=0.905) or adult exposure (F_6.28_ = 1.109; p=0.382) (**Supplemental Figure 1**).

### 3.3 Perinatal exposure to KTZ or DES disrupts the hypothalamic transcriptome

Because gestational and lactational exposure to DES and KTZ delayed vaginal opening and maturation of GnRH secretion, we hypothesized that the transcriptional activity of the glial and neuronal network governing GnRH secretion, in the mediobasal hypothalamus (MBH) and in preoptic area (POA), would be sensitive to perinatal exposure to KTZ or DES. To explore this hypothesis, RNA-seq was performed on MBH or POA total RNA obtained from females at different developmental time points (PND 22, 42 and 90) after perinatal exposure to the three doses of KTZ or DES (5 biological replicates per group).

**Figure 3:**
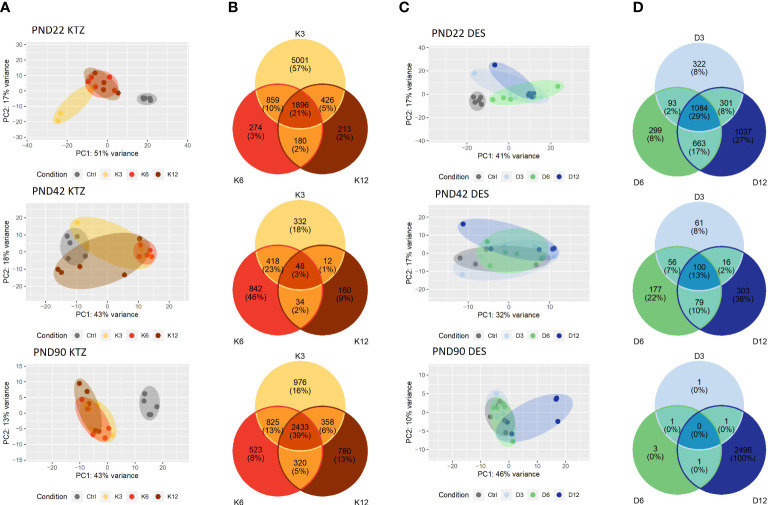
Effects of perinatal exposure to KTZ or DES on the mediobasal hypothalamus transcriptome. **(A, C)** Principal activity component (PCA) plot showing the dispersion of the transcriptome of MBH samples on PND 22, 42 or 90 after perinatal exposure to vehicle (control) or DES (D3: 3; D6: 6; or D12: 12 µg/kg bw/day) or KTZ (K3: 3; K6: 6; or K12: 12 mg/kg bw/day). Panels **(B, D)**: Venn diagrams representing the number of differentially expressed transcripts based on an adjusted p-value of 0.05 for all 3 doses of KTZ **(B)** or DES **(D)** compared to controls. N=5 samples/group.

In the MBH, the principal component analysis (PCA plot, **Figure 3**) indicated that the transcriptional profiles of control and EDC exposed samples were readily distinguishable at specific ages. At PND22, the three doses of KTZ or DES were clearly separated from the control group. The first two components explained 68% of total point variance for KTZ (**Figure 3A**) and 58% of total variability for DES (**Figure 3C**). At PND42, there were no clear clustering between EDC samples and control. At PND90, the three groups exposed to KTZ, or the highest dose of DES were clearly separated from the controls (**Figure 3A, C**).

We conducted differential expression analysis with an adjusted p-value threshold of 0.05 on each set of raw expression measures. The MBH appeared to be very sensitive to perinatal exposure to DES or KTZ before puberty as the mRNA expression of 1896 hypothalamic genes was significantly affected by all three doses of KTZ (**Figure 3B**); and 1084 genes by all three doses of DES (**Figure 3D**) on PND22. On PND 42, 48 genes were affected by all 3 doses of KTZ and 100 genes by the 3 doses of DES. On PND90, 2433 genes are affected by the three doses of KTZ while only the highest dose of DES altered a high number of genes (2496). The list of DEG was deposited on the Figshare repository under accession link: https://figshare.com/s/778fbb8f785c97d5d9ee.

**Figure 4:**
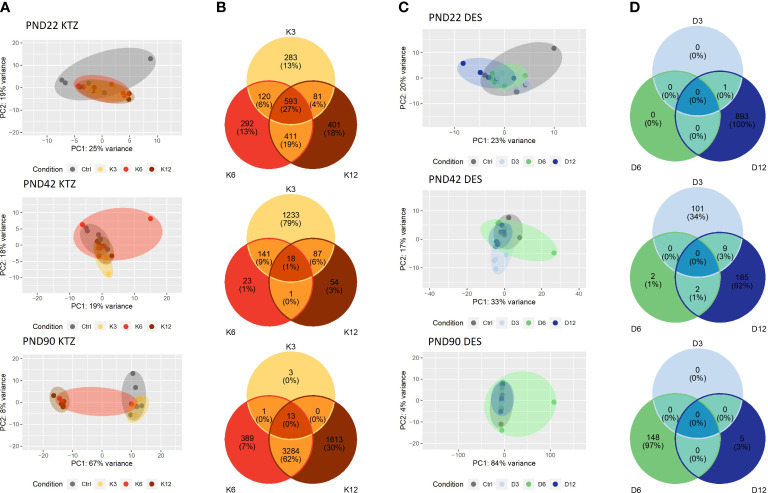
Effects of perinatal exposure to KTZ or DES on the Preoptic area (POA) transcriptome. **(A, C)** Principal activity component (PCA) plot showing the dispersion of the transcriptome of POA samples on PND 22, 42 or 90 after perinatal exposure to vehicle (control) or DES (D3: 3; D6: 6; or D12: 12 µg/kg bw/day) or KTZ (K3: 3; K6: 6; or K12: 12 mg/kg bw/day). Panels **(B, D)**: Venn diagrams representing the number of differentially expressed transcripts based on an adjusted p-value of 0.05 for all 3 doses of KTZ **(B)** or DES **(D)** compared to controls. N=5 samples/group.

RNA-seq of the preoptic area (POA) indicated a milder effect of perinatal exposure to KTZ or DES on the transcriptome compared to MBH. The principal component analysis (PCA plot, **Figure 4C**) revealed no distinction between the control and the DES clusters leading to an absence of genes affected by all three doses of DES. After KTZ exposure, 593 genes were commonly affected by the three doses at PN22 but only 18 at PND42 (**Figure 4B**). At PND 90, 13 genes were affected by all 3 doses of KTZ but the intermediate and the high doses shared 3284 affected genes (**Figure 4B**).

### 3.4 Perinatal exposure to KTZ or DES affects common hypothalamic pathways in the MBH before puberty

**Figure 5:**
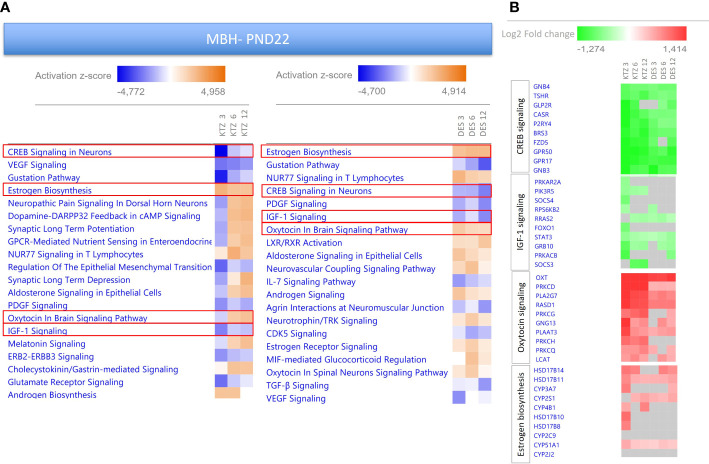
Enriched pathways differentially expressed in the MBH of PND22 females after perinatal exposure to KTZ or DES compared to controls. **(A)** Heatmaps comparing the most enriched canonical pathways for the 3 doses of KTZ or DES were generated by ranking pathways according to the Z-score and applying threshold and filters as described in the material and methods section. The z-score predicts whether a canonical pathway or diseases and biological functions are increased (positive z-score, orange) or decreased (negative z-score, blue) in accordance with the experimental dataset. Darker colors indicate higher absolute z-scores. **(B)** Heatmaps representing the Log2FC of the 10 most affected up- or downregulated genes for 4 selected pathways. Red or green colors indicate a significant change in expression (adjusted p-value < 0.05). Grey indicates non-significant changes.

To investigate possible biological interactions of differentially regulated genes in the MBH at PN22, RNA-seq datasets were imported into the Ingenuity Pathway Analysis Tool. Ingenuity Pathway Analysis (IPA) was used to predict enriched canonical pathways and their activation or inhibition. Heatmaps comparing the most enriched canonical pathways for the 3 doses of KTZ or DES were generated by ranking pathways according to the Z-score and by applying thresholds and filters as described in the material and methods. A Z-score >2 was defined as the threshold for significant activation, whilst Z-score <−2 was defined as the threshold for significant inhibition (55, 56). The heatmaps comparing the 20 most enriched canonical pathways for the 3 doses of KTZ and the three doses of DES are shown in **Figure 5**. Several important canonical pathways were shared among the three doses of each EDC. *“Estrogen biosynthesis”, “Creb signaling in Neurons”, “IGF-1 signaling”* and *“Oxytocin in brain signaling*” are critical pathways for reproductive function. The prediction of their activation or inhibition were also similar between the doses of each EDC and between both EDCs. As illustrated by the heatmaps (**Figure 5B**) representing the Log2-fold change of the 10 most up- or downregulated genes for these 4 selected pathways, it appears that the consequences of KTZ and DES exposure led to similar transcriptional effects. For instance, the oxytocin transcript (*Oxt*) was upregulated by the 3 doses of KTZ but also by the three doses of DES. The subcellular representations of these 4 pathways are provided in **Supplemental Figures 2–5**.

The heatmaps comparing the 20 most enriched canonical pathways after exposure to the 3 doses of KTZ in the POA at PND22 (**Supplemental Figure 6**), also revealed critical pathways for reproductive function such as “*Creb signaling pathway*” and *“oxytocin in brain signaling*” that are predicted to be downregulated. The two pathways that are predicted to be activated in the POA involve PPAR signaling. Notably, the Log2FC of the differentially expressed genes (DEG) in the POA are smaller than DEG changes observed in the MBH.

### 3.5 PPAR, a common upstream target of KTZ and DES

Using IPA upstream regulator analysis to identify potential upstream signals that may be driving changes in gene expression in the MBH, “PPARγ” was identified for all doses of KTZ and DES when compared with controls, with a z-score > 2.

**Figure 6:**
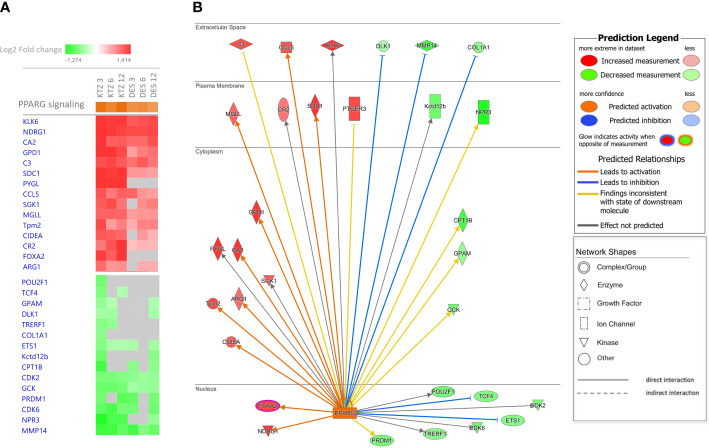
Ingenuity Pathway Analysis (IPA) identified PPARγ as upstream regulators. **(A)** The heatmap provides the top 15 up- and downregulated genes regulated by PPARγ in the MBH after exposure to the 3 doses of KTZ or DES. **(B)** The subcellular localization of the DEG products after KTZ 3 mg/kg bw/day exposure in the MBH at PND22, represents the direct interactions between the upstream regulator (PPARγ) and the downstream upregulated (labelled in red) or downregulated (labelled in green) genes. The upregulation of 10 and the downregulation of 5 of those genes are consistent with PPARγ activation (orange and blue arrows respectively). Two upregulated genes and 5 downregulated genes are inconsistent with PPARγ activation.

**Figure 6** provides a heatmap identifying the top 15 up- and downregulated genes regulated by PPARγ in the MBH after exposure to the 3 doses of KTZ or DES. The comprehensive gene list is provided in **Supplementary Table 2**. As an illustration, the subcellular localization of the DEG products after KTZ 3mg/kg.d exposure in the MBH at PND22 is shown in **Figure 6B**. This figure represents the direct interactions between the upstream regulator (PPARγ) and the downstream top upregulated (labelled in red) or downregulated (labelled in green) genes. The upregulation of 10 and the downregulation of 5 of those genes are consistent with PPARγ activation (orange and blue arrows respectively). Two upregulated genes and 5 downregulated genes are inconsistent with PPARγ activation.

The IPA tool, Tox Lists, helps to link experimental data to toxicological responses by providing sets of molecules that are known to be involved in a particular type of toxicity. In the Ingenuity Tox lists, “*PPARα/RXRα activation*” is systematically found among the most significant pathways for each EDC dose compared to control (KTZ 3_p-value =2.29x107; KTZ 6_p-value =5.88x103, KTZ 12_p-value =2.43x102; DES 3_p-value =4.89 x102; DES 6_p-value =2.45 x102; DES 12_p-value =5.88 x103) with an overlap of 15 to 48% between the genes of this pathway and the DEG of each EDC dose. This toxicological activation is systematically found in the 4 canonical pathways, affected in the same direction by three doses of each EDC in the MBH on PD22 (“*Estrogen biosynthesis”, “Creb signaling in Neurons”, “IGF-1 signaling”* and *“Oxytocin in brain signaling*”) (**Supplemental Figures 2 to 5**).

### 3.6 Perinatal exposure to KTZ or DES affects mRNA expression of genes critical for puberty onset

As we previously shown, perinatal exposure to the three doses of DES and the intermediate dose of KTZ led to a significant delay in vaginal opening without affecting bodyweight (21). This observation is consistent with the delayed maturation of GnRH secretion induced by exposure to the two highest doses of KTZ and DES (**Figure 2**). We screened the PND 22 MBH RNA-seq data set for all genes known to be associated with hypogonadotropic hypogonadism or familial self-limited delayed puberty (59, 60) in order to identify potential target genes involved in delayed puberty caused by KTZ or DES. **Table 1** indicates the level of expression of genes known to be mutated in pathological conditions leading to a delay of puberty and significantly affected by at least 2 doses of KTZ on PND22 in the MBH. The comprehensive gene list is provided in **Supplemental Table 3**. Among them, *Igsf10*, for which loss-of-function mutations lead to delayed puberty, is downregulated by all doses of DES and KTZ at PND22 (61).

**Table 1:** Genes known to be mutated in pathological conditions leading to a delay of puberty and significantly affected by at least 2 doses of KTZ at PND22 in the MBH.

	KTZ 3		KTZ 6		KTZ 12		DES 3		DES 6		DES 12	
	Log2FC	Padj	Log2FC	Padj	Log2FC	Padj	Log2FC	Padj	Log2FC	Padj	Log2FC	Padj
*Sema7a*	1,09	3,57E-07	1,13	2,83E-07	1,25	1,00E-08	0,73	8,37E-03	0,75	5,41E-03	0,77	3,10E-03
*Nsmf*	1,59	4,16E-09	1,17	3,79E-05	1,15	6,26E-05	0,65	2,34E-02	0,79	3,84E-03	0,77	4,32E-03
*Fezf1*	-0,82	1,06E-03	-0,60	3,73E-02	-0,70	1,47E-02	-0,56	7,21E-02	-0,36	2,58E-01	-0,57	4,63E-02
*Igsf10*	-1,10	1,35E-06	-0,83	7,86E-04	-0,93	1,40E-04	-0,62	1,39E-02	-0,83	3,55E-04	-0,83	2,49E-04
*Sema3a*	-1,12	2,97E-05	-0,62	5,10E-02	-1,00	6,17E-04	-0,83	1,45E-03	-0,54	5,32E-02	-0,80	1,84E-03
*Pnpla6*	-0,45	2,54E-04	-0,31	2,66E-02	-0,13	4,53E-01	-0,10	5,44E-01	-0,21	1,26E-01	-0,24	6,67E-02

In addition, we screened the RNA-seq data set for genes known to regulate GnRH neuron activity directly or indirectly and compared their level of expression after perinatal exposure to the three doses of KTZ and DES in the MBH at PND22 (**Table 2**). The data indicates that a high number of genes involved in the transcriptional and epigenetic control of GnRH secretion are very consistently affected by the 3 doses of DES and KTZ at PN22. Notably, *Mkrn3*, known to act as a brake on GnRH secretion and puberty onset (62), was downregulated by all doses of DES and KTZ. Glutamatergic and GABAergic pathways appeared also very sensitive to KTZ and DES. The glutamatergic receptor, *Grm3*, was upregulated by all the doses of KTZ and DES while seven subunits of GABA receptor were significantly affected by the 3 doses of KTZ and by some doses of DES. In addition, the mRNA expression of other receptors able to modulate reproductive function, such as *Ghr, Gpr37, Esrra* or *Tshr*, were also altered in the same direction by all the doses of KTZ and nearly all doses of DES. The two neuropeptides, arginine vasopressin (*Avp*) and oxytocin (*Oxt*) synthesized in magnocellular neurons located mainly in the supraoptic (SON) and paraventricular (PVN) nuclei of the hypothalamus, appear to be upregulated by all the doses of KTZ and DES. The expression of *Npw*, neuropeptide identified to play a role in regulating energy homeostasis during postnatal development (63), was also strongly downregulated by all doses of KTZ and DES.

**Table 2:** Genes known to regulate GnRH activity and their level of expression (Log2FC) after perinatal exposure to the three doses of KTZ and DES in the MBH at PND22 and PND90.

	KTZ							DES					
	P22				P90			P22			P90		
Pathway	Gene	K3	K6	K12	K3	K6	K12	D3	D6	D12	D3	D6	D12
Transcriptional and epigenetic control of puberty and reproduction	*Cbx7*	0.493***	0.614***	0.630***	0.704***	0.754***	0.695***	0.578***	0.529***	0.540***			0.400**
	*Dnmt3a*	-1.263***	-0.804**	-0.694*	-0.975***	-0.928***	-0.892***	-0.651*	-0.595*	-0.824**			-0.417**
	*Dnmt3b*	-1.030***	-0.959**	-0.556			-0.478*	-0.794**	-0.604*	-0.924***			
	*Ezh1*	0.335*	0.481**	0.569***	0.451***	0.519***	0.620***	0.500***	0.424***	0.421***			0.357***
	*Ezh2*	-0.429*	-0.560**	-0.544**	-0.788***	-0.843***	-0.896***	-0.685***	-0.361*	-0.591***			-0.418*
	*Mkrn3*	-0.686**	-0.918***	-0.778***	-0.922***	-0.926***	-1.043***	-0.808***	-0.777***	-0.709**			
	*Gata3*	0.871**	0.814*	1.270***									
	*Sema7a*	1.092***	1.130***	1.246***			0.502**	0.725**	0.748**	0.775**			
	*Prc1*				-0.479**	-0.612***	-0.528**						
	*Sirt2*		-0.354*	-0.393*	-1.031***	-1.114***	-1.136***	-0.415**	-0.328*				-0.427*
	*Per3*	-0.932***	-0.598**	-0.421*	-0.287*	-0.342*	-0.401**	-0.528*	-0.433*	-0.657**			-0.394***
Gabaergic signaling	*Gabbr1*	0.154*	0.191*	0.213**	0.135*			0.235*					
	*Gabbr2*	0.476*	0.629**	0.750***			0.324*	0.520*					
	*Gabra4*	1.234***	1.372***	1.336***					0.555*				
	*Gabra5*	-0.462***	-0.352***	-0.515***				-0.424***	-0.467***	-0.425***			
	*Gabrd*	2.143***	2.011***	2.138***			0.307*	0.372***	0.445***	0.353***			
	*Gabre*	-1.711***	-1.509***	-1.555***	-0.782***	-0.786***	-0.856***	-0.897**	-1.102***	-1.022***			-0.421*
	*Gabrq*	-1.287***	-0.739*	-0.833*						-0.863**			
	*Slc32a1*												
	*Abat*				0.393***	0.439***	0.287**						0.256**
	*Gnai2*				0.322***	0.279***	0.210**						
	*Trak2*	0.955***	0.715***	0.607**	0.414*	0.421*		0.489**	0.524**	0.758***			0.391**
Calcium signaling	*Cacna1i*	-0.716***			-0.310**	-0.236*	-0.311**						-0.344**
	*Cacna1h*	-0.781***	-0.778***	-0.524*	-0.401**	-0.336*	-0.356*		-0.501*	-0.504*			-0.481***
	*Cacna2d3*	0.370*	0.493*	0.515*									
	*Cacna2d4*	0.944**	0.785*							0.618*			
	*Cacnb1*	-0.550***	-0.556***	-0.434**	-0.372***	-0.402***	-0.320**	-0.325*	-0.311*	-0.353*			-0.286***
	*Cacng3*	0.239*	0.319*	0.285*			0.331*		0.334*				
	*Cacng4*				-0.220***	-0.296***	-0.217***						-0.152*
Glutamatergic signaling	*Grid2ip*	1.938***	2.068***	2.232***				0.652***	0.753***	0.725***			
	*Grik2*	-0.579***	-0.334*	-0.405*	-0.338**				-0.391*	-0.420*			
	*Grm3*	1.168***	1.365***	1.308***	0.658***	0.606***	0.802***	1.210***	1.198***	1.219***			0.474**
	*Grm4*	0.755**	0.693**	0.993***		-0.434*							
	*Neto1*	-1.163***	-0.526*	-0.703**	-0.539***		-0.389*	-0.543*		-0.692**			
	*Slc17a7*	1.832***	1.744***	1.877***				0.388***	0.544***	0.511***			
	*Adcy6*	-0.745***	-0.527***	-0.420**	-0.479***	-0.448***	-0.414***	-0.449**	-0.499**	-0.469**			-0.369***
	*Itpr1*		0.631**	0.587**	0.414**	0.503**	0.612***						0.409***
	*Slc38a3*		-0.248**		-0.252**	-0.349***	-0.427***			-0.264**			
Neuropeptides and receptors controlling reproductive function	*Crhr2*	-1.019***	-0.962***	-1.043***				-0.588*	-0.735**				-0.360*
	*Esr1*	-1.130***	-0.571*	-0.860***									
	*Esrra*	0.854***	0.516*	0.580**					0.591*	0.519*			
	*Ghr*	-0.846***	-0.561**	-0.701***			-0.346*	-0.533*	-0.541**	-0.666***			
	*Gpr37*	0.345*	0.452**	0.625***	0.266*		0.447***	0.585***	0.491***	0.521***			
	*Rxra*	-0.539***	-0.397**	-0.325*						-0.369*			
	*Rxrg*	-0.509***	-0.570***	-0.585***					-0.414*				
	*Tshr*	-0.935***	-0.851***	-1.008***				-0.794**	-0.720**	-0.765**			
	*Oxt*	2.509***	2.014***	1.651***	0.427*			1.300***	1.271***	1.369***			
	*Pdyn*	-0.624***	-0.708***	-0.775***					-0.498*	-0.697**			
	*Pgf*	0.900***	0.507*	0.477*	0.607***	0.459**	0.384*	0.509*	0.607**	0.572**			
Control of metabolism	*Adora1*	0.777***	0.710***	0.672***				0.473*	0.482*	0.503*			0.306*
	*Agrp*	-0.620*	-1.027***	-1.032***			-0.584**						
	*Arg1*	1.023***	0.725***	0.769***					0.568*	0.563*			
	*Avp*	1.994***	1.337***	1.129***	0.869***		0.779***	0.847***	0.789***	0.895***			
	*Car2*	1.520***	1.103***	1.132***	0.783***	0.655***	0.805***	1.024***	1.030***	1.211***			0.476**
	*Cck*	1.897***	1.486***	1.529***				0.363*	0.603**	0.435*			
	*Gpr12*	0.464*	0.547*	0.549*					0.529*	0.547*			
	*Gpr17*	-1.488***	-1.207***	-1.109***	-2.211***	-2.306***	-2.182***	-1.035***	-1.096***	-1.101***			-0.380*
	*Igfbp5*	0.579***	0.715***	0.697***	0.701***	0.587***	0.718***	0.837***	0.750***	0.572***			0.472**
	*Insig1*	0.628***	0.527***	0.575***			0.344*	0.501**	0.444**	0.467**			
	*Mc3r*	-0.529*	-0.666**	-0.606**					-0.502*	-0.621**			
	*Npw*	-3.104***	-3.317***	-3.315***	-1.996***	-1.898***	-2.049***	-2.525***	-2.524***	-2.524***			-0.426**
	*Npy2r*	-1.257***	-0.684*	-0.806**						-0.679*			
	*Nr5a1*	-1.278***	-1.333***	-0.969***	-0.465*			-0.761*		-0.809**			-0.413*
	*Stat3*	-0.619***	-0.491**	-0.440*				-0.405*	-0.470**	-0.571***			

Upregulated (font color po ay red) or Downregulated (font color po ay green) genes /*p<0,05 ; **p<0,01 ; ***p<0,001.

### 3.7 Consequences of exposure to KTZ or DES on adult transcriptome

**Figure 7:**
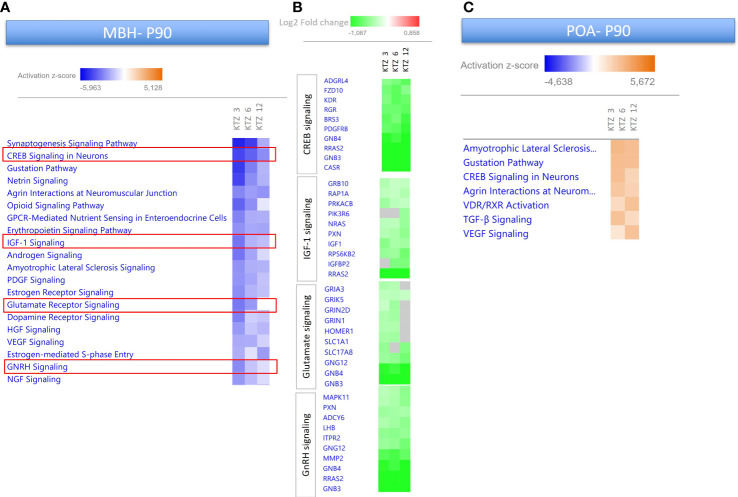
Enriched pathways differentially expressed in the MBH and POA of PND90 females after perinatal exposure to KTZ compared to controls. **(A)** Heatmaps comparing the most enriched canonical pathways for the 3 doses of KTZ were generated by ranking pathways according to the Z-score and applying threshold and filters as described in the material and methods section. The z-score predicts whether a canonical pathway or diseases and biological functions are increased (positive z-score, orange) or decreased (negative z-score, blue) in accordance with the experimental dataset. Darker colors indicate higher absolute z-scores. **(B)** Heatmaps representing the Log2FC of the 10 most downregulated genes for 4 selected pathways. Green color indicates a significant change in expression (adjusted p-value < 0.05). Grey indicates non-significant changes. **(C)** Heatmaps comparing the most enriched canonical pathways in the POA for the 3 doses of KTZ were generated by ranking pathways according to the Z-score.

As depicted in **Figures 3**, **4**, perinatal exposure to KTZ but not DES appeared to significantly affect hypothalamic (MBH and POA) transcriptional activity in adulthood. The heatmaps presented in **Figure 7** compares the 20 most enriched canonical pathways in the MBH or POA after perinatal exposure to the 3 doses of KTZ. IPA mainly predicted a strong inhibition of the most affected pathways in the MBH and a moderate activation of a limited number of pathways in the POA. Several enriched pathways appeared conserved between PND 22 and PND 90 in the MBH. “*Creb signaling in Neurons”, “IGF-1 signaling”* and *“Glutamate receptor signaling*” were still predicted to be downregulated. The comparison also revealed “*GnRH signaling pathways*” among the top downregulated pathways.

Several DEGs at PND22 in the MBH showed similar alterations at PND90 after KTZ exposure. Three PolyComb genes important for the epigenetic regulation of reproductive genes (64), *Cbx7, Ezh1* and *Ezh2*, were all affected by the three doses of KTZ at both ages. The glutamatergic receptor, *Grm3*, and the ϵ subunit of GABAergic receptor were still upregulated and downregulated, respectively, at PND90. The expression of the neuropeptide, *Npw* was also strongly downregulated by all doses of KTZ at PND90.

Finally, we assessed by RT-qPCR the mRNA expression of six genes after pubertal and adult exposures to KTZ and DES. These six genes were chosen because they were differentially affected by the perinatal exposure to most of the doses of KTZ and DES at PND22 and PND90. Only *Dnmt3* mRNA expression was affected by pubertal or adult exposure to one dose of KTZ (**Supplemental Figure 7**).

## 4 Discussion

This study assessed the consequences of exposure to two EDCs with different mode of action, ketoconazole (steroidogenesis inhibitor) and diethylstilbestrol (strong estrogen), on the hypothalamic transcriptome governing GnRH release in female rats. To consolidate the study, multiple doses and different windows of exposure were tested. We found that perinatal exposure to KTZ and DES delayed the maturation of GnRH secretion in female rats which is consistent with the delay in vaginal opening we previously reported for the same animals (21). Our results underline the exquisite sensitivity of the mediobasal hypothalamus to developmental exposure to EDCs. In particular, the transcription of crucial actors of the GnRH network was affected by the developmental exposure to both estrogenic and anti-androgenic compounds.

### 4.1 GnRH release is sensitive to EDC exposure

We have previously shown that developmental exposure to estrogenic EDCs can disrupt the hypothalamic control of puberty (19, 20, 65). Our hypothalamic incubation model retains the pulsatile characteristics of GnRH secretion which are defined by a physiological acceleration before the onset of puberty (51). This model is thus well suited to detect the central effects of EDCs on GnRH secretion *ex vivo*. We previously documented that delayed vaginal opening caused by developmental exposure to EDCs can be explained by a slowdown of GnRH pulsatile release before puberty. Exposure to DES at 1 µg/kg.day during the first 5 postnatal days led to a slower release of GnRH at PND 25 and to delayed vaginal opening (19). The same observation was made after exposure to 25 ng/kg.day of BPA during the first 5 or 15 postnatal days while a higher dose of 5 mg/kg.day accelerated maturation of GnRH secretion and advanced puberty (20). In this study, we observed that perinatal exposure to 12 µg/kg.day DES and 12 mg/kg.day KTZ slowed down GnRH secretion consistent with the reported delayed vaginal opening (21). The prepubertal period was the most sensitive in detecting the impact of EDCs on GnRH release as the impact of DES and KTZ on GnRH secretion was less pronounced at later ages (PND 42 and 90). This indicates that the mechanisms that trigger the activation of the GnRH pulse generator at puberty onset may be more sensitive to KTZ or DES than the mechanisms maintaining GnRH secretion later in life (66, 67). The activation of GnRH release around puberty results from a loss in trans-synaptic inhibition together with a rise in excitatory input (34). This is explained by a shift in expression of puberty activating and inhibitory genes. Our results suggest that early exposure to KTZ or DES disrupt the shift in expression of key inhibitory or excitatory factors which are responsible for GnRH secretion acceleration at puberty. In addition, our data identified the perinatal period as a critical window of sensitivity, as exposure to DES or KTZ during that period led to an alteration of GnRH pulsatility while pubertal or adult exposures had no impact on GnRH pulse frequency in our model. However, we cannot exclude that such later exposure could affect other parameters such as the amplitude of GnRH pulses *ex vivo* which is physiologically increased during the afternoon of the proestrus (68).

### 4.2 EDC exposure disrupts extrinsic GnRH pulse generator

We show here that a very high number of hypothalamic genes is affected by perinatal exposure to KTZ and DES when studied during the prepubertal period (PND 22). This illustrates the high sensitivity of the hypothalamic transcriptional regulation to EDC exposure. The cell bodies of GnRH neurons are located in the POA. Their projections cross the MBH to end in the median eminence where GnRH is released in a pulsatile manner. Compelling data indicate that the pulse generator is not intrinsic to GnRH neurons, but rather is extrinsic and located within the arcuate nucleus and MBH (32, 67, 69). In our study, the MBH transcriptome appeared more sensitive to KTZ and DES exposure than the POA, as shown by the higher number of DEGs and the broad amplitude of expression changes. KNDy (expressing Kisspeptin, Neurokinin B and Dynorphin) and GABAergic neurons appear to be major actors of the GnRH pulse generator (32, 67, 69). Recent work by Herbison demonstrated that the inhibitory GABAergic signalization on GnRH dendrons requires GABA_B_ receptor and voltage-gated calcium channels (VGCC)(70). In our study, the expression of two subunits of GABA_B_ receptor and two subunits of VGCC, *Cacna2d3* and *Cacng3*, are upregulated by all doses of KTZ in the MBH at PND22, which is consistent with a delay in the activation of the extrinsic GnRH pulse generator and slowdown of GnRH pulsatile secretion. The opposite observation is made for the P22 downregulation of *Mkrn3*, that has been recently demonstrated to prevent puberty initiation, at least in part, by repressing the transcription of the genes coding for Kisspeptin and Neurokinin B (62). Finally, we cannot exclude that the delay in GnRH secretion maturation is due to the disruption of embryonic migration or development of GnRH neurons by DES or KTZ. *Igsf10* mutations are associated with abnormal GnRH neuronal migration resulting in delayed puberty (61). Notably, in our study, *Igsf10* is downregulated by all doses of KTZ and DES at PND22. Additionally, epigenetic factors driving fgf8-dependent GnRH neuron development such as *Dnmt* and *Ezh2* (71), were downregulated by the developmental exposure to KTZ.

### 4.3 Biomarkers of KTZ and DES exposure

To identify potential biomarkers of exposure, we focused on DEGs and pathways that were affected by all 3 doses of KTZ and DES*. Ingenuity pathway analysis^®^(IPA)* is a powerful analysis and interpretation tool built on the comprehensive and manually selected content of the QIAGEN Knowledge Base. Using IPA, we identified pathways predicted to be up or down regulated by perinatal exposure to KTZ and DES. At PND 22, the top pathway predicted to be activated by DES and KTZ was “*estrogen biosynthesis*”. The role of neuroestrogens in the regulation of GnRH and puberty is still incompletely understood. Terasawa et al. showed that neuroestradiol, locally synthesized in the hypothalamus, could be a component of the central inhibition of GnRH release before puberty in monkeys (72). Estradiol (E_2_) levels in the median eminence are higher before than during puberty onset when GnRH release begins to increase (73). Because neuroestrogens appear to halt puberty, the activation of this pathway seems consistent with the delay of maturation of GnRH release observed in our model of exposure.

The “*Creb signaling pathway*” was systematically identified as downregulated by the three doses of KTZ in the MBH at PND22 and PND90 and in the POA at PND22. DES exposure similarly led to a downregulation of this pathway in the MBH at PND22. cAMP response element-binding protein (Creb) is a transcription factor which binds to cAMP response element (CRE) of the promoter of its target genes. As illustrated in **Supplemental Figure 2**, Creb can act as a second messenger upon activation of glutamate receptors, growth factor receptors or an intracellular Ca2+ influx (74, 75). GnRH neurons express Creb which acts as a mediator of E2 negative feedback (76). Creb has already been reported to be a target of anti-androgenic EDC in the hippocampus (77–79) and the testes (80).

The “*Igf‐1 (insulin-‐like growth factor 1) signaling pathway*” was another pathway constantly affected by perinatal exposure to KTZ and DES. Igf1 is a metabolic signal activating and enhancing GnRH secretion at the time of puberty 81). IGF1 effect on GnRH neurons is mediated by glial production of PGE2 (81) and by neurokinin B release by KNDys neurons (82). Its expression is sensitive to EDCs, as it is upregulated in the arcuate nucleus after perinatal exposure to BPA or DES (83). Recent data indicates that precocious puberty in female rats following peripubertal exposure to 5 mg/kg/day of DEHP was associated with an upregulation of the hypothalamic Igf-1/Pi3k/Akt/mTor pathway. Inhibition of Igf-1R and mTor prevented the action of DEHP by decreasing Kiss-1, Gpr54, and GnRH expression (84). These reports support Igf1 as an interesting biomarker of EDC impact on the hypothalamic network.

We identified *Oxt* and *Avp* as potential biomarkers, as they were among the genes most affected by perinatal exposure to KTZ and DES at PND 22 and PND 90. The pathway “*Oxytocin in brain signaling*” was also identified by IPA analyses. Oxytocin appears to play a key role in pubertal onset and ovulation in several species. In rats, oxytocin facilitates female pubertal development through a mechanism involving Pge2 release by glial cell and increases the expression of several actors of the GnRH network including kisspeptin (85–87). In fish, studies suggest a modulation of the hypothalamo-pituitary-gonadal axis by oxytocin (88). In women, oxytocin is involved in the physiological regulation of LH through the ovulatory cycle (89). Hypothalamic oxytocin appears to be sensitive to EDC exposure. In our previous study, a perinatal exposure to a mixture of estrogenic and anti-androgenic EDCs led to a transgenerational downregulation of *Oxt* mRNA expression in the hypothalamus of prepubertal female rats (65). In the present study, we observed an opposite effect. *Oxt* was upregulated after both KTZ and DES perinatal exposure. These changes appear to contradict the delayed maturation of GnRH secretion and puberty. but could be seen as reactive rather than causal as previously seen for BPA effects on hypothalamic expression of enzymes involved in GABA synthesis (20). Thus, *Oxt* appears as a sensitive target, and a potential biomarker, of developmental exposure to EDCs.

Our results indicate that PPARγ could be one of the upstream regulators driving the changes in gene expression caused by KTZ and DES in the MBH. PPARγ is a nuclear receptor and a transcription factor. It regulates the expression of genes responsible for adipocyte differentiation, placental differentiation, lipid and glucose homeostasis, control of inflammatory responses and steroidogenesis (90). PPARγ is also expressed by hypothalamic AgRP and NPY neurons which are involved in the control of energy balance in mice and rats (91, 92). Those neurons send projections to GnRH neurons and regulate their activity (reviewed in (93)). One could hypothesize that PPARγ expressed by AgRP/NPY neurons could mediate EDC effects on GnRH secretion. However, the role of PPARγ as a metabolic sensor regulating GnRH activity remains to be determined. Several studies indicate that PPARγ expression and activity are sensitive to EDCs. It has been identified as a major mediator of the obesogenic effects of phthalates or bisphenol A, in adipocytes (94, 95). PPARγ is also expressed in the ovary where its activity is induced by mono-(2-ethylhexyl) phthalate (MEHP)(96) or Perfluoroalkyl and polyfluoroalkyl substances (PFAS) (97). All together, these data suggest that PPARγ could also be a potent effector and biomarker of EDC effects on female reproduction and lend further support to a previously developed putative AOP that proposes a PPARγ-mediated reduction in aromatase to explain irregular ovarian cycling and impaired fertility in adult females (*AOP-Wiki*).

It should be considered that our transcriptome data were obtained from whole MBH or POA, both of which are made up of various cell populations. Thus, it can be difficult to separate changes to general cellularity (a shift in cell populations) from bona fide changes in gene transcription. This is a prevailing challenge for RNA-seq analysis of heterogenous tissues. Future studies including single-cell RNA-seq could be used to identify specific cell populations sensitive to EDC exposure by deconvolution approaches. Nevertheless, our data suggests that a large proportion of DEGs represent changes in transcription, as we did not observe any obvious trend of all (or most) genes specific to certain cell types were moving in one direction; either up- or down-regulated.

### 4.4 Mode of action of ketoconazole

KTZ is mostly known to inhibit various P450 cytochrome enzymes involved in steroidogenesis and thereby interfering with both androgen and estrogen synthesis (39, 40). However, the role and expression of aromatase in the brain during puberty onset has been poorly studied. Aromatase (*Cyp19a1*) mRNA expression in the hypothalamus is very low during the infantile period (98, 99) and almost undetectable in the POA at P21 (100). Recent studies using modified mice where EGFP transcription is coupled to the physiological activation of Cyp19A1, show that aromatase is well expressed in the medial preoptic area of the hypothalamus during embryonic life (101) and during adulthood (102). However, infantile, and pubertal periods have not been studied with this technology. This suggests that KTZ exposure during gestation and lactation could impact aromatase transcription or activity during the embryonic period. During this period, aromatase plays a crucial role in sexual differentiation of the brain. The classical view of brain sexual differentiation in mammals holds that sex differences mostly depend on the production of testosterone by the testes (103). Depending on the species, some of the effects of testosterone result from the action of estradiol derived from aromatization of testosterone locally in the male brain. For this reason, any compound which interferes with estrogen-dependent processes, as estrogenic or anti-androgenic compounds, in the developing brain can disrupt the sexual differentiation and consequently also the programming of puberty and reproductive function. Thus, we cannot exclude that the hypothalamic disruption is indirect and due to an altered release of peripheral steroids as KTZ can inhibit steroidogenic P450 enzymes in the adrenal cortex (104, 105) and gonads (105, 106). A recent review also reported that azole fungicides may affect all levels of the hypothalamo-pituitary-adrenal & gonadal axes in fish (107).

## 5 Conclusions

GnRH secretion and the hypothalamic transcriptome of female rats are sensitive to perinatal exposure to both DES and KTZ. Our results point toward similar transcriptional consequences after exposure to a steroidogenesis inhibitor (KTZ) or an estrogenic compound (DES). In particular, perinatal exposure to KTZ strongly impacted hypothalamic gene expression before puberty with consequences persisting until adulthood. Several enriched pathways as well as differentially expressed genes that were affected at PND 22 were still altered at PND 90. These pathways should be explored further to identify biomarkers for future EDC testing strategies and be part of future standard information requirement in risk assessment of chemicals.

## Data availability statement

The original contributions presented in the study are publicly available. This data can be found here: 10.6084/m9.figshare.21842124.

## Ethics statement

The animal study was reviewed and approved by Danish Animal Experiments Inspectorate; authorization number 2015-15-0201-00553.

## Author contributions

DF: Conceptualization, Formal analysis, Data interpretations, Writing – Original draft, Writing – review & editing. HJ: Conceptualization, Data interpretations, Writing – review & editing. DL-R: Formal analysis, Data interpretations, Writing – review & editing. AL: Data interpretations, Formal analysis, Writing – review & editing. QT: Technical support, Formal analysis. JB: Conceptualization, Writing – review & editing, Funding acquisition. SC: Conceptualization, Writing – review & editing. TS: Conceptualization, Writing – review & editing, Funding acquisition. A-SP: Conceptualization, Data interpretations, Writing – Original draft, Writing – review & editing, Funding acquisition. All authors contributed to the article and approved the submitted version.
