# Supplementary material for: Perinatal exposure to the fungicide ketoconazole alters hypothalamic control of puberty in female rats
Source: Front Endocrinol (Lausanne). 2023 Apr 3;14:1140886. doi: 10.3389/fendo.2023.1140886 (PMC10108553; doi:10.3389/fendo.2023.1140886)
Supplement: Supplementary file 1 [file DataSheet_1.docx]

Supplementary Material

**Perinatal exposure to the fungicide ketoconazole alters hypothalamic control of puberty in female rats**

**
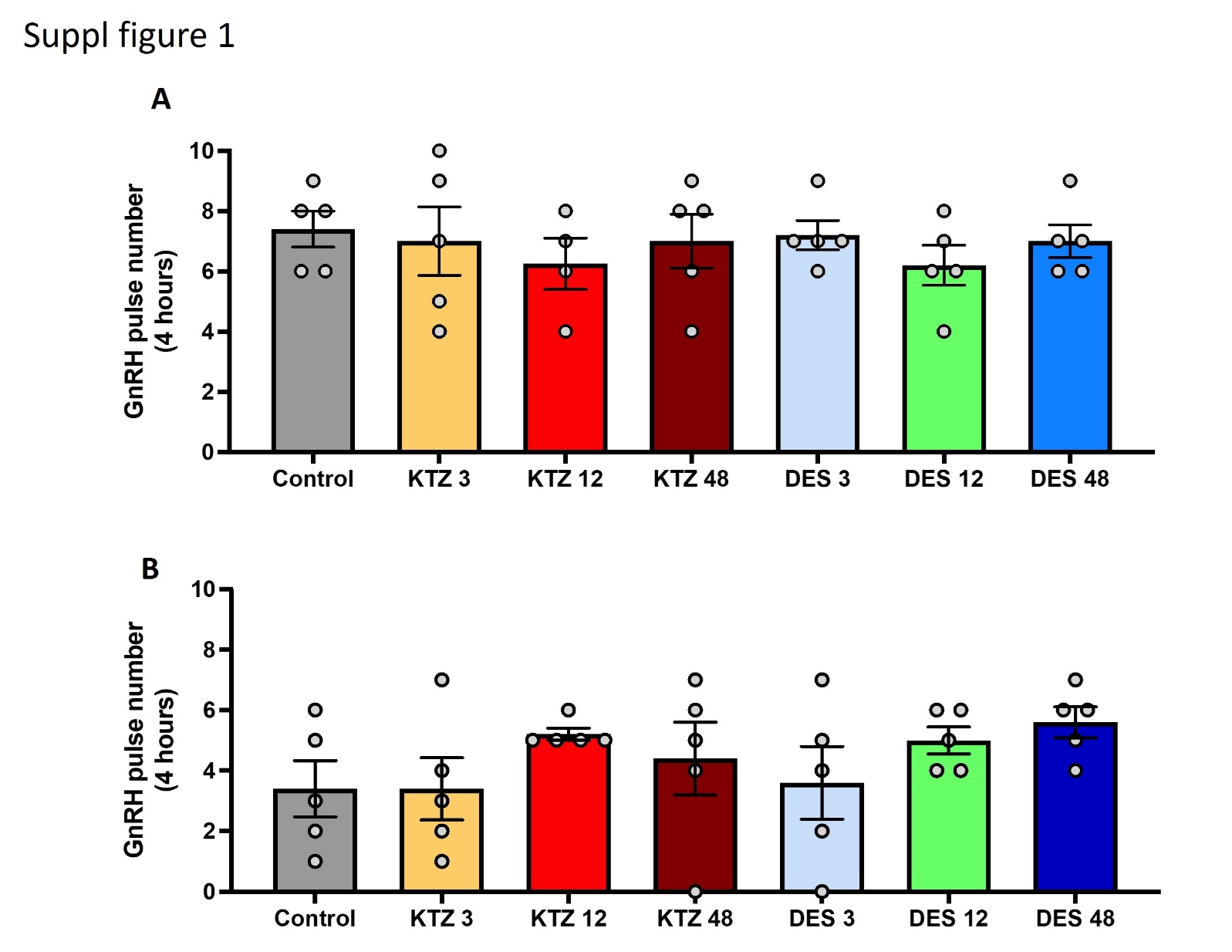
**

**Suppl figure 1** : Effects of pubertal (A) or adult (B) exposure to KTZ and DES on pulsatile GnRH secretion from hypothalamic explants *in vitro*. (A) Average GnRH pulse numbers/4 hours were measured *ex vivo* using hypothalamic explants obtained from female rats on PND 50 after peripubertal exposure to vehicle (control), DES (3; 12; or 48 µg/kg bw/day) or KTZ (3; 12; or 48 mg/kg bw/day). (B) Average GnRH pulse numbers/4hours were measured *ex vivo* using hypothalamic explants obtained from female rats on PND 90 after adult exposure to DES or KTZ. Data are mean (n=4-6/group) +/- SEM.


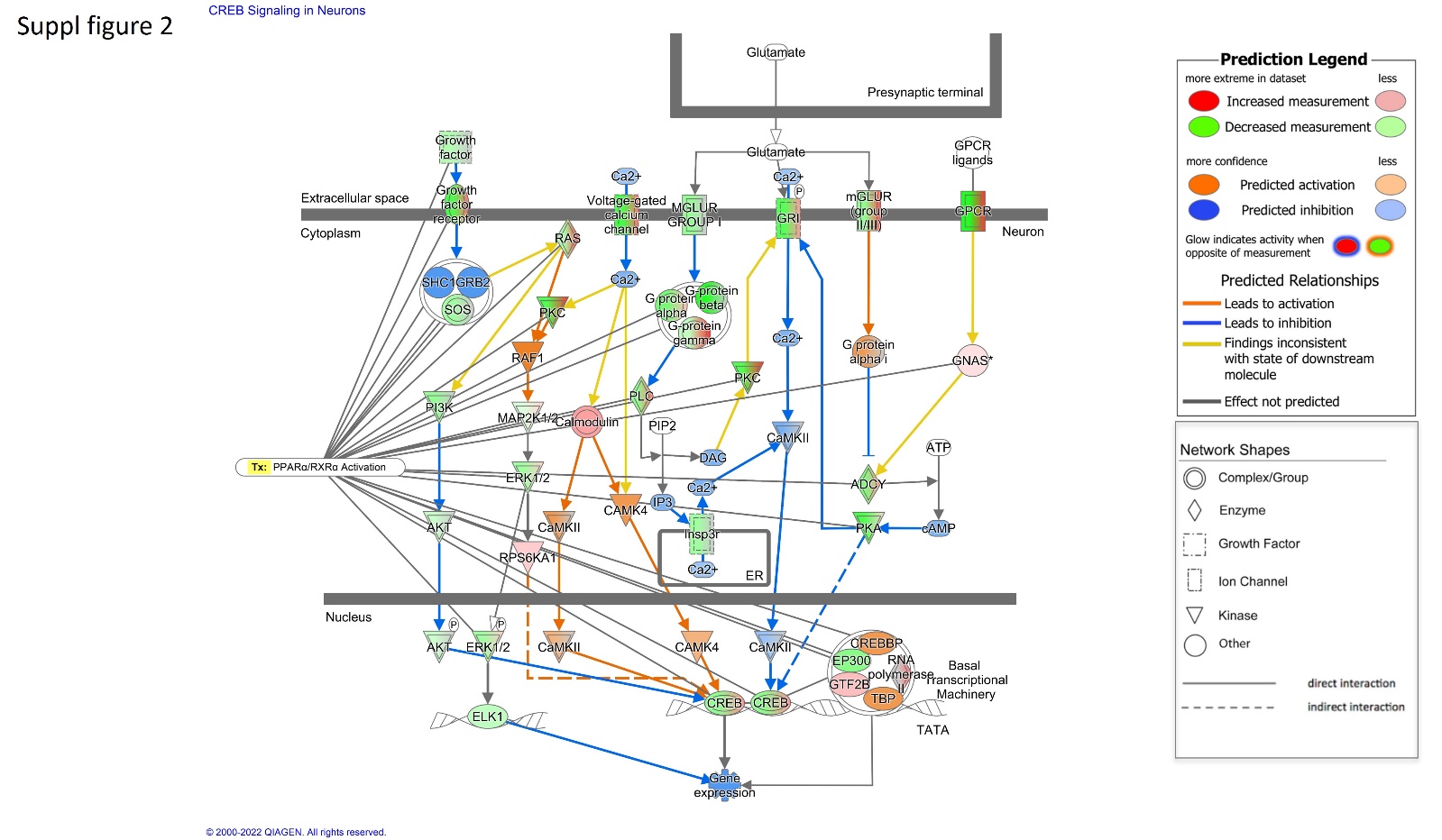


**Suppl figure 2**: CREB signaling pathway identified by IPA. The CREB signaling pathway was identified as an enriched pathway differentially expressed in the MBH of PND22 females after perinatal exposure to KTZ or DES compared to controls. The figure illustrates here the subcellular localization of the DEG products after KTZ 3 mg/kg bw/day exposure in the MBH at PND22. All up-regulated DEGs are labelled in red while down-regulated DEG are labelled in green. The Ingenuity Tox list indicates “PPARα/RXRα activation” as a pathway involved in the regulation of a high proportion of the DEG belonging to the CREB pathway.

**
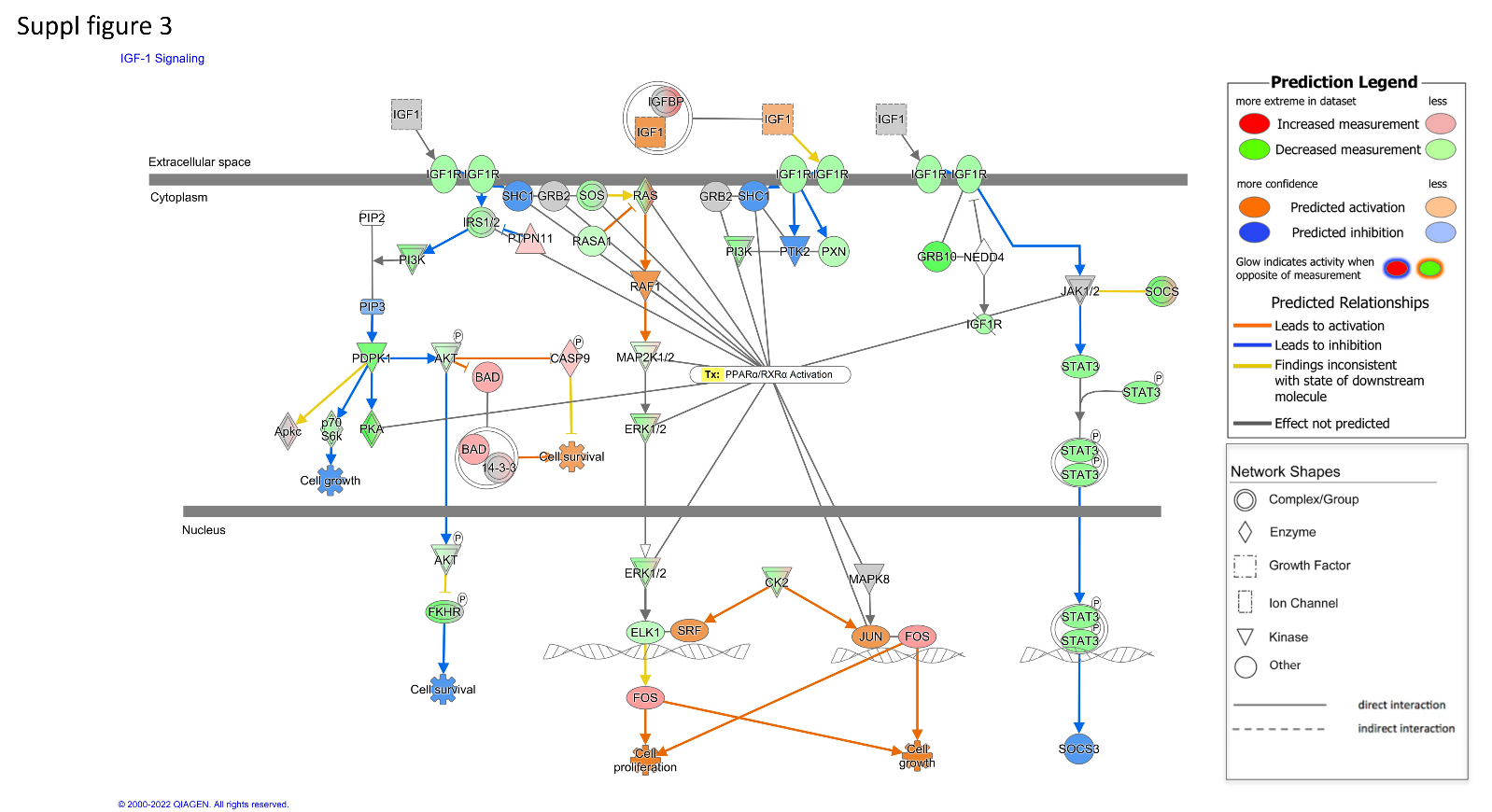
**

**Suppl figure 3**: IGF-1 signaling pathway identified by IPA. The IGF-1 signaling pathway was identified as an enriched pathway differentially expressed in the MBH of PND22 females after perinatal exposure to KTZ or DES compared to controls. The figure illustrates here the subcellular localization of the DEG products after KTZ 3 mg/kg bw/day exposure in the MBH at PND22. All up-regulated DEGs are labelled in red while down-regulated DEG are labelled in green. The Ingenuity Tox list indicates “PPARα/RXRα activation” as a pathway involved in the regulation of a high proportion of the DEGs belonging to IGF-1 pathway.

**
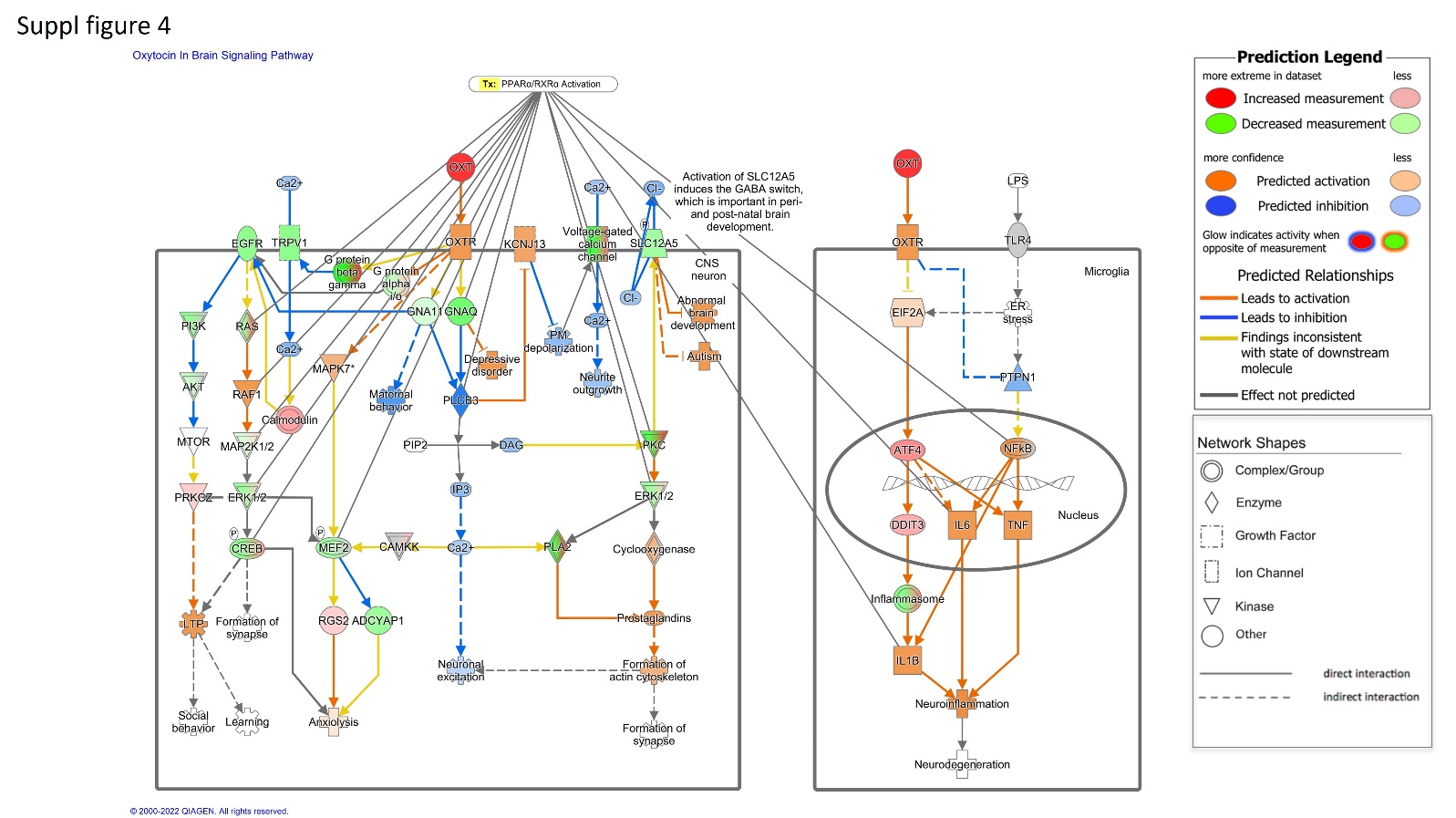
**

**Suppl figure 4**: Oxytocin in Brain signaling pathway identified by IPA. The Oxytocin in Brain signaling pathway was identified as an enriched pathway differentially expressed in the MBH of PND22 females after perinatal exposure to KTZ or DES compared to controls. The figure illustrates here the subcellular localization of the DEG products after KTZ 3 mg/kg bw/day exposure in the MBH at PND22. All up-regulated DEGs are labelled in red while the down-regulated DEG are labelled in green. The Ingenuity Tox list indicates “PPARα/RXRα activation” as a pathway involved in the regulation of several DEG belonging to *Oxytocin in Brain signaling* pathway.

**
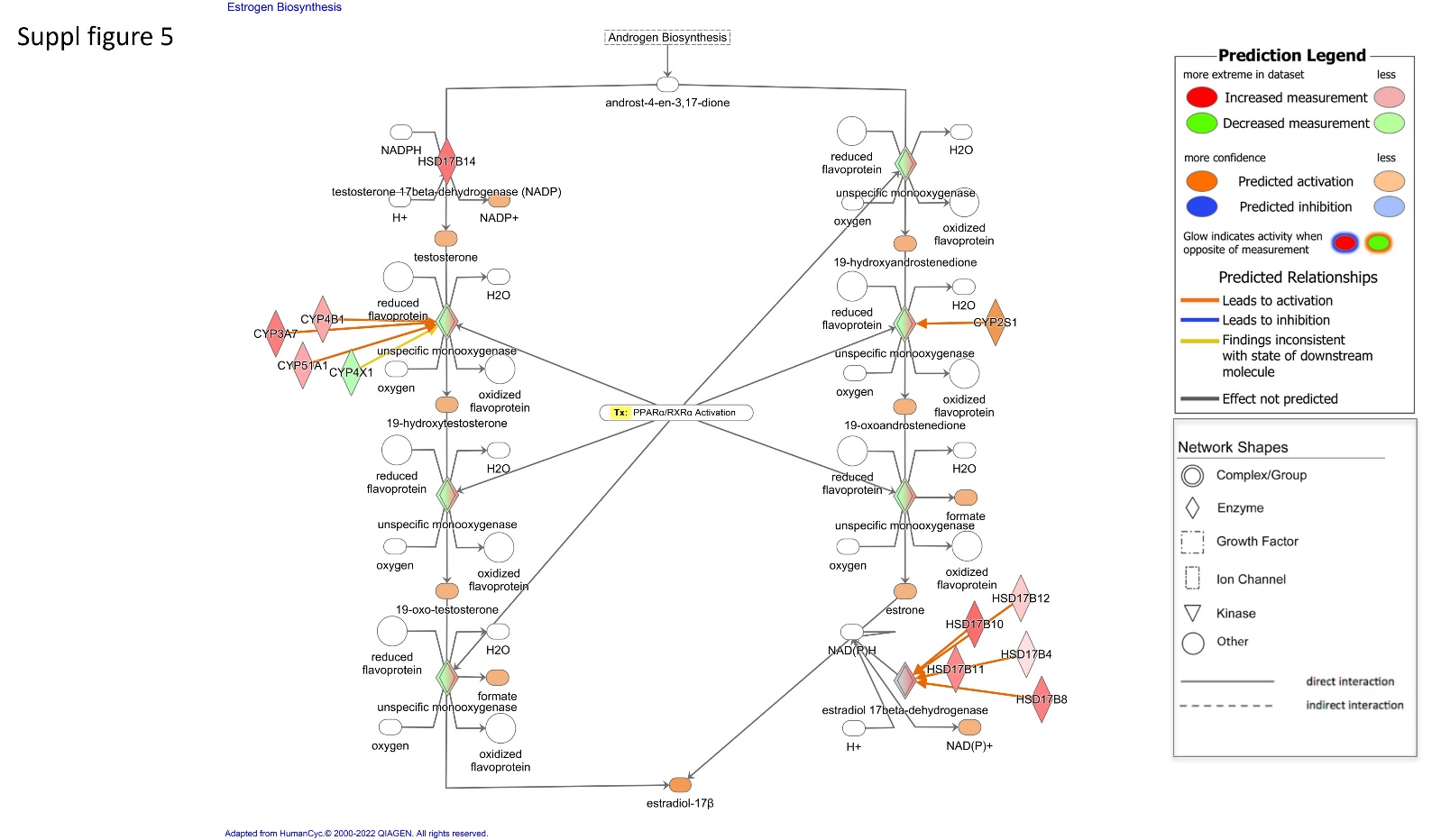
**

**Suppl figure 5**: Estrogen biosynthesis pathway identified by IPA. The Estrogen biosynthesis pathway was identified as an enriched pathway differentially expressed in the MBH of PND22 females after perinatal exposure to KTZ or DES compared to controls. The figure illustrates here the subcellular localization of the DEG products after KTZ 3 mg/kg bw/day exposure in the MBH at PND22. All the up-regulated DEGs are labelled in red while the down-regulated DEG are labelled in green. The Ingenuity Tox list indicates “PPARα/RXRα activation” as a pathway involved in the regulation of some DEGs belonging to Estrogen biosynthesis pathway.

**
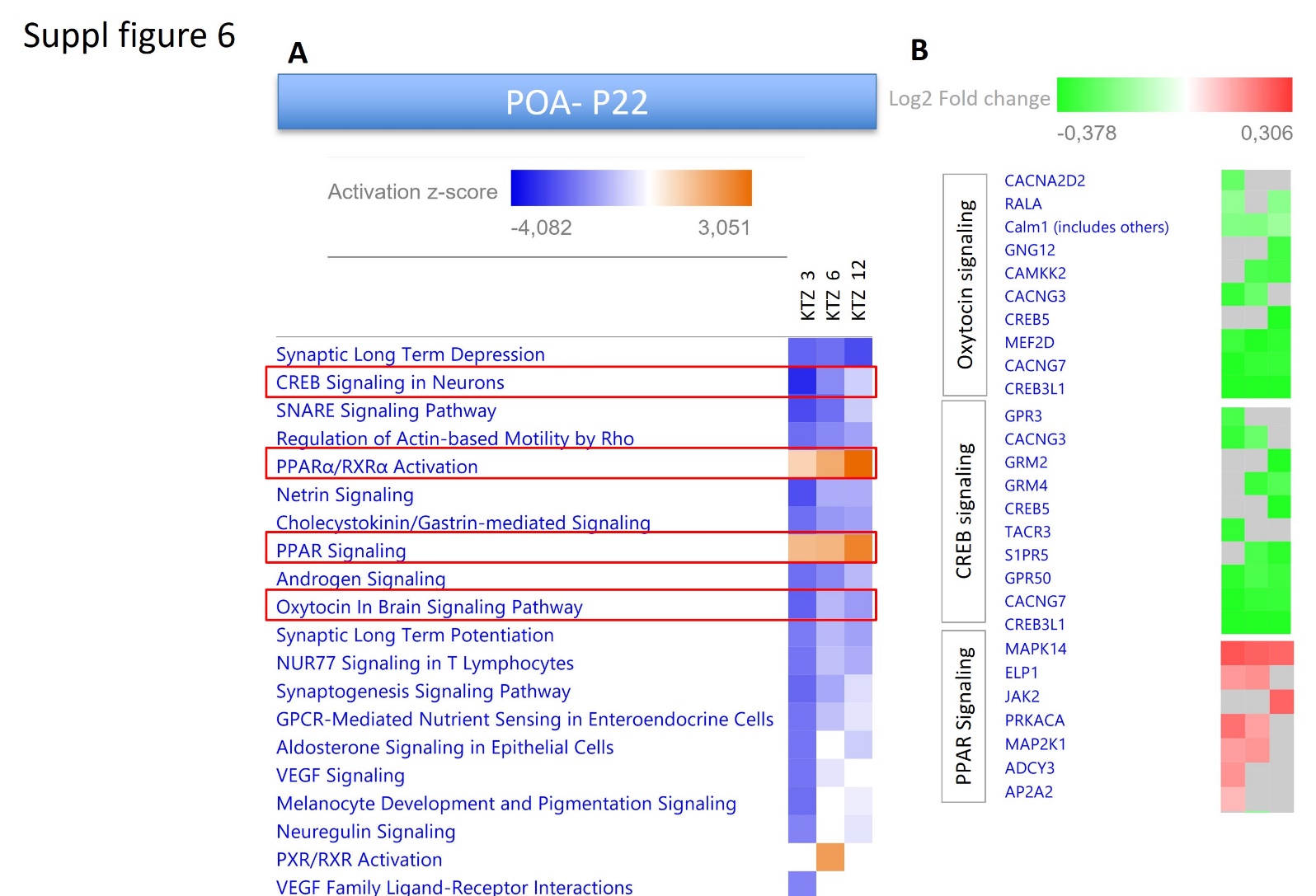
**

**Suppl figure 6:** Enriched pathways differentially expressed in the POA of PND22 females after perinatal exposure to KTZ compared to controls. (A) Heatmaps comparing the most enriched canonical pathways for the 3 doses of KTZ were generated by ranking pathways according to the Z-score and applying threshold and filters as described in the material and methods section. The z-score predicts whether a canonical pathway or diseases and biological functions are increased (positive z-score, orange) or decreased (negative z-score, blue) in accordance with the experimental dataset. Darker colors indicate higher absolute z-scores. (B) Heatmaps representing the Log2FC of the 10 most down- or upregulated genes for 3 selected pathways. Green and red colors indicate a significant change in expression (adjusted p-value < 0.05). Grey indicates non-significant changes.

**
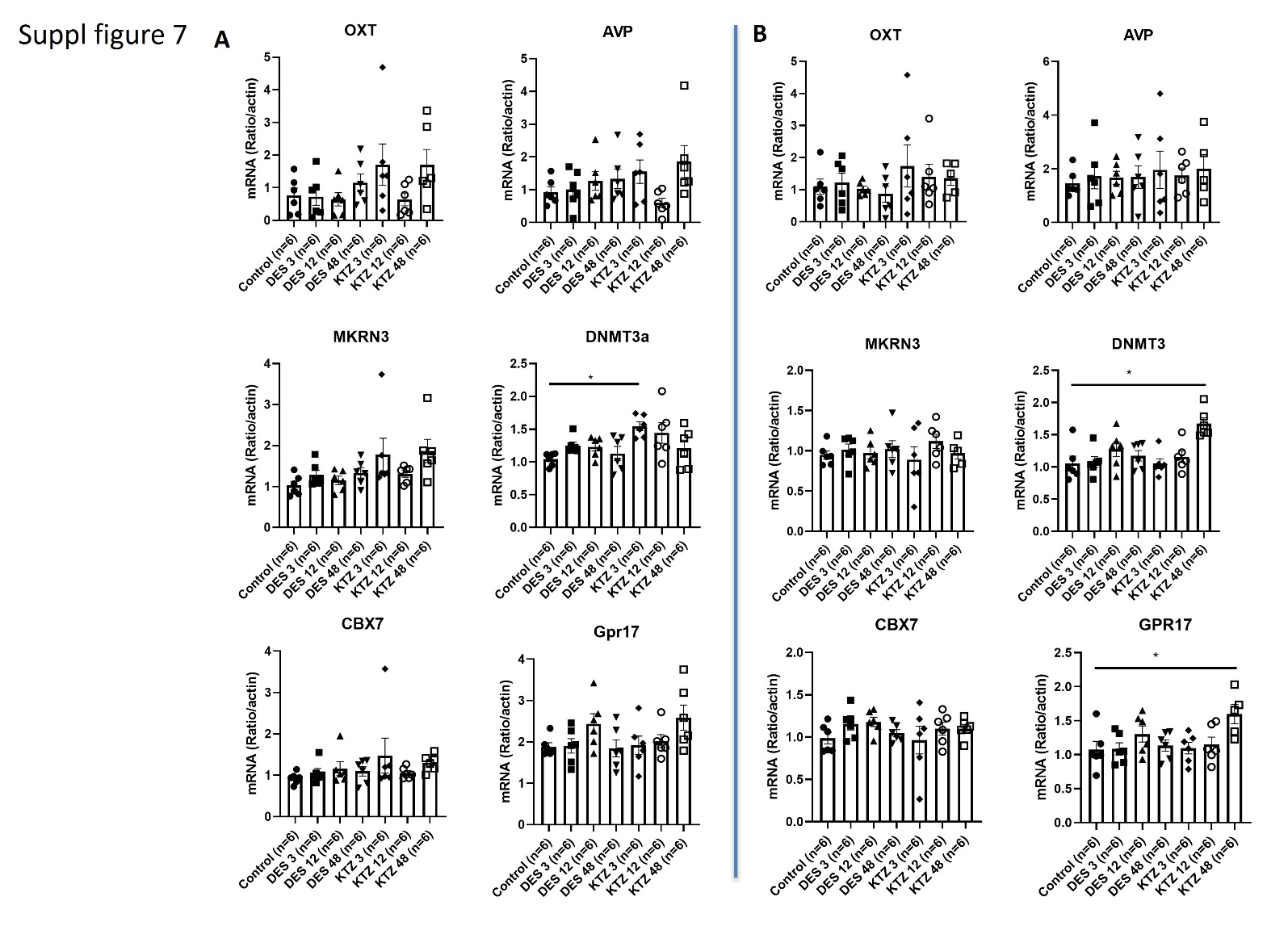
**

**Suppl figure 7:** mRNA expression of hypothalamic genes after pubertal (A) and adult (B) exposure to KTZ and DES. The six studied genes were DEG after perinatal exposure to most of the doses of KTZ and DES at PND22 and PND90. After pubertal and adult exposure to KTZ and DES, only DNMT3 mRNA expression was affected by pubertal and adult exposure to one dose of KTZ. Data are mean (n=6) +/- SEM; *p < 0.05 versus control at the same age.

Supplemental table 1 : Primer sequences

| Genes | Primer sequence |
| --- | --- |
| *Oxt* | Fw : GCTGCCAGGAGGAGAACTAC Rv :ATCATCACAAAGCGGGCTCA |
| *Avp* | Fw : AGCGATGAGAGCTGCGTG  Rv : CTGTACCAGCCTAAGCAGCA |
| *Mkrn3* | Fw : GCCTAGGTCACTGCCCATTT  Rv : ACGCTTCCCTCTCGAAACTG |
| *Dnmt3* | Fw : ACACGGCAGAATAGCCAAGT  Rv : GGGAAGCCAAACACCCTTTC |
| *Cbx7* | Fw : ATGGAGCTGTCAGCCATAGG  Rv : ATTCAACTTTGCCCTTCCGC |
| *Gpr17* | Fw : TTCCTCACCTGCATCAGTGC  Rv : CACTAGCAGTGGGGCCATAG |

Supplemental table 2  : Lis of up- and downregulated genes regulated by PPARγ in the MBH after exposure to the 3 doses of KTZ or DES.

Supplemental table 3 : Comprehensive list of genes known to be mutated in pathological conditions leading to a delay of puberty and their level of expression at PND 22 in MBH after KTZ and DES perinatal exposure.
